# Survival kinetics of *Listeria monocytogenes* on chickpeas, sesame seeds, pine nuts, and black pepper as affected by relative humidity storage conditions

**DOI:** 10.1371/journal.pone.0226362

**Published:** 2019-12-11

**Authors:** Joelle K. Salazar, Vidya Natarajan, Diana Stewart, Quincy Suehr, Tanvi Mhetras, Lauren J. Gonsalves, Mary Lou Tortorello

**Affiliations:** 1 Division of Food Processing Science and Technology, U. S. Food and Drug Administration, Bedford Park, Illinois, United States of America; 2 Institute for Food Safety and Health, Illinois Institute of Technology, Bedford Park, Illinois, United States of America; University of Hong Kong, HONG KONG

## Abstract

Nuts and seeds have been increasingly associated with recalls due to contamination with *Listeria monocytogenes*. Storage of these food commodities occurs at various relative humidity (RH) conditions for months or years. The objective of this study was to assess *L*. *monocytogenes* survival on four commodities representing dried legumes, seeds, and spices categories: chickpeas, sesame seeds, pine nuts, and black pepper kernels. Inoculated products at 10 log CFU/g were stored for 180 days (6 months) at 25°C and different relative humidity (RH) levels: 25% (low), 45% (ambient), and 75% (high). After 180 days at 25% RH, *L*. *monocytogenes* populations decreased to 2.67–6.59 log CFU/g; the highest survival of the pathogen was observed on pine nuts and sesame seeds with decay rates of -0.014± 0.001 log CFU/g per d. Significantly greater population reductions on all products were observed during storage at 45 and 75% RH. At 45% RH, *L*. *monocytogenes* levels decreased to 1.90–6.36 log CFU/g. On chickpeas and black pepper stored at 75% RH, the pathogen population decreased to below the limit of enumeration (1 log CFU/g) yet were still detected via enrichments. The lowest survival of *L*. *monocytogenes* occurred at 75% RH on black pepper with a decay rate of -0.058±0.003 log CFU/g per d. Overall, regardless of RH level, the ability of the products to support survival of the pathogen may be expressed in the following order: pine nuts > sesame seeds > chickpeas > black pepper. The results of this study can aid in understanding how *L*. *monocytogenes* survives on dried legumes, seeds, and spices, and the data can contribute to the risk assessment of this pathogen.

## Introduction

Nuts, seeds, spices, and dried legumes are low water activity (a_w_, <0.85) foods and have often been implicated in outbreaks of illness associated with *Salmonella enterica* and pathogenic *Escherichia coli* [1–5]. Although no illnesses associated with *Listeria monocytogenes*-contamination of these commodities have been reported, recent recalls of nuts and seeds due to contamination of *L*. *monocytogenes* have been associated with almonds [6, 7], cashews [7, 8], macadamia nuts [9], pine nuts [10], walnuts [11–15], and sunflower seeds [16]. *L*. *monocytogenes* is ubiquitous in the environment, and these products can become contaminated at various stages throughout production including harvesting, processing, distribution, and storage. After processing, nuts and seeds can be stored prior to distribution for long periods of time. For example, raw nuts, depending on the type, may be stored frozen for 1–10 years, refrigerated for up to 18 months, or at ambient temperature for 3–6 months [17, 18]. *L*. *monocytogenes* has also been shown to survive in low a_w_ food products for long periods of time [19–25].

Long-term survival kinetics data of *L*. *monocytogenes* on nuts are available for almonds [22], peanuts [23], pecans [23], in-shell pistachios [22], and in-shell walnuts [24, 25]. *L*. *monocytogenes* was detectable for one year on raw peanuts and pecans inoculated at approximately 4–5 log CFU/g and stored at -24, 1, or 22°C [23]. The pathogen was also detectable for one year on raw almonds and in-shell pistachios inoculated at approximately 4–5 log CFU/g and stored at -19, 4, and 24°C [22]. Overall, the *L*. *monocytogenes* population did not significantly decrease on the almonds or pistachios when stored frozen or at refrigeration. However, at 24°C, the population of the pathogen decreased at a rate of -0.71 and -0.86 log CFU/g per month on almonds and pistachios, respectively. Similar decreases were also observed at 24°C for *S*. *enterica* and *E*. *coli* O157:H7. No information exists pertaining to *L*. *monocytogenes* survival on dried legumes, seeds, or spices during storage.

In addition to storage temperature, the relative humidity (RH) in which these commodities are stored impacts the survival of pathogens. Studies have shown that *S*. *enterica* survives better on nuts when stored at lower RH and a_w_ levels [26–28]. At 25°C and 0.37 a_w_ storage, *S*. *enterica* decreased 1 log CFU/g on hazelnuts and pine nuts after 24 and 52 weeks, respectively [26]; whereas at 0.54 a_w_, the same decrease was observed on hazelnuts and pine nuts after only 9 and 16 weeks, respectively. Except for a thorough examination of survival during drying of fermented sausage [29], little information is available on how *L*. *monocytogenes* survives on low a_w_ food commodities stored at different RH levels.

This study aimed to determine the survival of *L*. *monocytogenes* on different low a_w_ food products at three RH levels: 25 (low), 45 (ambient), and 75% (high). Four different commodities from legume, seed, and spice categories were chosen: chickpeas, pine nuts, sesame seeds, and black pepper kernels. Results of this study can aid in understanding the impact of RH storage on *L*. *monocytogenes* survival on these low a_w_ food commodities.

## Materials and methods

### Legumes, seeds, and spices

At least two different lots of chickpeas (legume; *Cicer arietinum* L.), sesame seeds (seed; *Sesamum indicum* L), pine nuts (seed; *Pinus pinea* L.), and black peppercorns (spice; *Piper nigrum* L.) were obtained in bulk from online retailers and stored sealed at 25°C for up to 2 weeks before use.

### Strains, culture conditions, and inoculum preparation

Spontaneous rifampicin resistant variants of *L*. *monocytogenes* strains 0806 (isolated from hummus), 3132 (isolated from avocado), 0352 (isolated from cream cheese), and ScottA (isolated from milk [30]) were obtained by successive culturing in Brain Heart Infusion broth (BHI; Thermofisher Scientific, Waltham, MA) with increasing concentrations of rifampicin, up to the final level of resistance at 200 μg/mL. Wild-type strains were obtained from the FDA Stock Culture Collection in Bedford Park, IL. All strains were cultured individually in BHI supplemented with 200 μg/mL of rifampicin and incubated at 37°C for 16–18 h. One hundred μL of each strain was plated onto each of 5 replicate BHI agar (BHIA; Thermofisher Scientific, Waltham, MA) plates and incubated at 37°C for 24 h. Cells were harvested by adding 1.5 mL of Butterfield’s Phosphate Broth (BPB; Thermofisher Scientific, Waltham, MA) to each plate and scraping with a sterile disposable culture spreader. Five replicate plates for each strain were harvested and combined into a 50-ml tube, resulting in approximately 40 ml of cocktail (inoculum level approximately 11 log CFU/mL). The four-strain cocktail was serially diluted and plated onto Plate Count Agar (PCA; Thermofisher Scientific, Waltham, MA) with 200 μg/mL rifampicin to verify initial inoculation levels.

### Inoculation of chickpeas, sesame seeds, pine nuts, and black peppercorns

Two hundred forty g of chickpeas, sesame seeds, pine nuts, and black pepper were individually placed into 3-L stomacher bags. Products were inoculated with 40 mL of the 11 log CFU/mL four-strain cocktail of *L*. *monocytogenes* resulting in approximately 10 log CFU/g. The bags were massaged by hand for 5 min to distribute the inoculum on the product evenly. Triplicate 1-g samples from each aliquot were taken for enumeration of initial *L*. *monocytogenes* inoculation levels. The inoculated samples were poured onto aluminum foil, spread into a single layer, and dried in a biosafety cabinet for 24 h. Uninoculated control samples were prepared similarly using BPB instead of inoculum and dried in a biosafety cabinet for 24 h.

### Storage of inoculated food products

After drying 24 h, the products were transferred to 3-L stomacher bags. Triplicate 1-g samples were taken from each bag to determine *L*. *monocytogenes* population levels after drying. Each product was aliquoted into 7-g portions and placed into sterile zip-lock bags (16 cm × 10 cm). All zip-lock bags were opened and stored at 25°C at 25, 45, or 75% RH. RH levels were maintained at 25±1, 45±1, or 75±1% using saturated solutions of potassium acetate, potassium carbonate, or sodium chloride, respectively, in desiccator cabinets (Secador Mini Desiccator Cabinets, Bel-Art-SP Scienceware, Wayne, NJ). Data loggers (OM-EL-USB-2-LCD, Omega Engineering, Norwalk, CT) were placed inside each desiccator cabinet to monitor RH level and temperature.

### Enumeration of *L*. *monocytogenes*

At each time point of 1, 7, 14, 21, 28, 60, 90, 120, and 180 d, one zip-lock bag for each product (containing 7 g) at each RH was removed from storage. *L*. *monocytogenes* was enumerated by adding 1 g from the zip-loc bag, in triplicate, to 9 mL of Buffered *Listeria* Enrichment Broth (BLEB; Oxoid, Hampshire, UK), serially diluted, and plated onto PCA with rifampicin. To lower the level of enumeration of the plate count assay, when necessary, 1 mL of the original dilution was plated over 3 agar plates for a level of enumeration of 1 log CFU/g. The remaining sample in BLEB was enriched for *L*. *monocytogenes* according to the FDA BAM procedure, with modifications to account for a 1-g enrichment [31]. Triplicate samples were analyzed for each time point and two independent experiments were conducted (n = 6).

### Water activity measurements

The water activity of 1-g samples was measured using a water activity meter (Aqualab 4TE, Decagon, WA). Triplicate samples were analyzed for each time point of 0, 1, 7, 28, 90, and 180 d.

### Statistical analysis and modeling survival data

Significant differences between the change in water activity for each product during storage were determined using Tukey’s adjusted ANOVA using GraphPad Prism version 7.04. Numerical linear regression slope estimates, confidence intervals, and significance for *L*. *monocytogenes* survival were calculated for each nut and seed type at each RH using MATLAB version 2018b (The MathWorks, Natick, MA) using the MATLAB statistics toolbox function “aoctool”. The population data acquired showed no significant non-linearity that could not be justified by experimental error (i.e. Akike information criteria showed that a linear model most accurately represented the data). Therefore, population decay rates were quantified based on log-linear kinetics and tested for statistical difference by analysis of covariance (ANCOVA) using product type and RH as categorical factors. Post hoc comparison was performed by Tukey-Kramer’s Honest Significant Difference Test of the estimated slopes and a p-value less than 0.05 was considered significant.

## Results and discussion

### Changes in a_w_ of chickpeas, pine nuts, sesame seeds, and black pepper during 180 d storage

The RH levels in which the inoculated food commodities were stored did not significantly change over 180 d storage. The initial a_w_ of the chickpeas, pine nuts, sesame seeds, and black pepper after inoculum addition and drying for 24 h were 0.381, 0.289, 0.287, and 0.395, respectively (Table 1). No differences were observed between initial a_w_ (directly out of the package) or the a_w_ after inoculation and 24 h drying of the commodities. During storage at 25% RH, the a_w_ of the chickpeas, sesame seeds, and black pepper did not significantly change over 180 d. The a_w_ of the pine nuts significantly increased after 1 d to 0.418 and the final a_w_ at 180 d was 0.396. Generally, for 45 and 75% RH storage, the a_w_ of all commodities significantly increased over time. The highest increase in a_w_ at 45% RH storage was observed for sesame seeds (0.287 at 0 d to 0.449 at 180 d). The a_w_ after 180 d storage at 45% for all commodities ranged from 0.447–0.449. At 75% RH, the highest increase in a_w_ was also observed for sesame seeds (0.287 at 0 d to 0.689 at 180 d). The a_w_ after 180 d storage at 75% for all commodities ranged from 0.667–0.689. Similar increases in the a_w_ of nuts during storage have been determined by other studies [22, 23]. The moisture content of pecans and peanuts increased (from 2 to 3% (<0.3 to >0.4 a_w_ [32]) and from 4 to 5.8%, respectively) over 1 year when stored at 22°C and 56±8% RH [23].

**Table 1:** Water activity (a_w_) values of pine nuts, chickpeas, sesame seeds, and black pepper stored at 25°C at 25, 45, or 75% relative humidity (RH).

Time (d)	Water activity (a_w_)			
	Pine nuts	Chickpeas	Sesame seeds	Black pepper
25% RH				
0	0.289±0.097 ^a^	0.381±0.010 ^a^	0.287±0.012 ^a^	0.395±0.049 ^a^
1	0.418±0.065 ^b^	0.398±0.058 ^a^	0.285±0.018 ^a^	0.378±0.037 ^a^
7	0.442±0.118 ^b^	0.385±0.036 ^a^	0.332±0.067 ^a^	0.385±0.013 ^a^
28	0.310±0.038 ^ac^	0.376±0.034 ^a^	0.283±0.031 ^a^	0.349±0.019 ^a^
90	0.368±0.019 ^bc^	0.355±0.008 ^a^	0.318±0.007 ^a^	0.362±0.014 ^a^
180	0.396±0.026 ^bc^	0.376±0.023 ^a^	0.315±0.019 ^a^	0.369±0.024 ^a^
45% RH				
0	0.289±0.097 ^a^	0.381±0.010 ^a^	0.287±0.012 ^a^	0.395±0.049 ^ab^
1	0.455±0.033 ^b^	0.433±0.051 ^ab^	0.301±0.020 ^ab^	0.407±0.032 ^ab^
7	0.386±0.013 ^bc^	0.421±0.027 ^ab^	0.378±0.047 ^c^	0.387±0.021 ^a^
28	0.370±0.027 ^c^	0.409±0.018 ^ab^	0.398±0.008 ^c^	0.397±0.027 ^ab^
90	0.434±0.011 ^bc^	0.443±0.016 ^b^	0.437±0.008 ^d^	0.440±0.006 ^b^
180	0.447±0.016 ^b^	0.449±0.010 ^b^	0.449±0.009 ^d^	0.447±0.012 ^bc^
75% RH				
0	0.289±0.097 ^a^	0.381±0.010 ^a^	0.287±0.012 ^a^	0.395±0.049 ^a^
1	0.468±0.038 ^b^	0.442±0.030 ^b^	0.298±0.025 ^a^	0.410±0.028 ^a^
7	0.475±0.019 ^b^	0.437±0.017 ^b^	0.394±0.015 ^b^	0.449±0.012 ^a^
28	0.531±0.018 ^b^	0.493±0.044 ^b^	0.552±0.005 ^c^	0.510±0.040 ^b^
90	0.622±0.011 ^c^	0.606±0.005 ^c^	0.634±0.008 ^d^	0.610±0.011 ^c^
180	0.670±0.026 ^c^	0.667±0.026 ^c^	0.689±0.020 ^e^	0.677±0.019 ^d^

Values are mean ± standard deviation (n = 6)

Different lowercase letters indicate significant difference between a_w_ in the same food product at the same RH level on different days

### Influence of RH level on the population dynamics of *L*. *monocytogenes*

**Fig 1:**
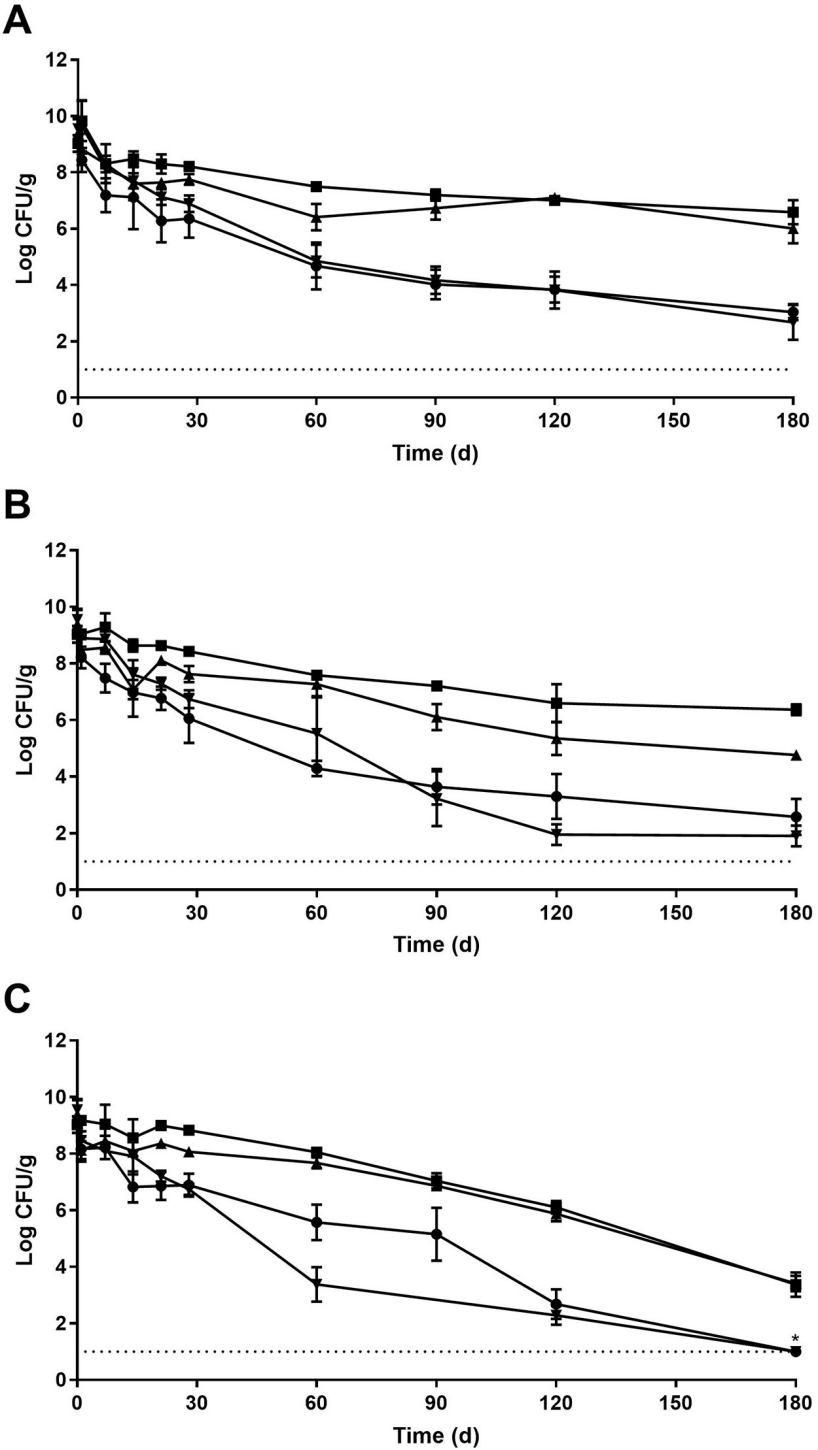
Survival of *L*. *monocytogenes* on pine nuts (■), sesame seeds (▲), black pepper (▼), and chickpeas (●) during storage for 180 d at 25°C and 25 (A), 45 (B), or 75% (C) relative humidity. Data points and error bars represent mean and standard deviation (n = 6). Dotted line indicates lowest level of enumeration (1 log CFU/g). * indicates that *L*. *monocytogenes* was not detected in enrichments.

After inoculation and drying for 24 h, the populations of *L*. *monocytogenes* on chickpeas, pine nuts, sesame seeds, and black pepper were 9.03±0.30, 9.04±0.30, 9.39±0.51, and 9.55±0.38 log CFU/g, respectively (Fig 1). At 25% RH (Fig 1A), the population of *L*. *monocytogenes* on chickpeas did not significantly decrease until 21 d of storage (6.28±0.74 log CFU/g) and continued to decrease over the course of 180 d; the final population at the end of storage was 3.03±0.29 log CFU/g. A similar population decrease was also observed on black pepper, where the *L*. *monocytogenes* level at 21 d was 7.12±0.28 log CFU/g and the final population after 180 d storage was 2.67±0.62 log CFU/g. The pathogen survived better on both sesame seeds and pine nuts at 25% RH. On sesame seeds, the *L*. *monocytogenes* population was significantly lower at 21 d to 7.63±0.13 log CFU/g and the ending population was 6.01±0.53 log CFU/g. For pine nuts, the *L*. *monocytogenes* level also did not significantly decrease until 21 d storage to 8.30±0.34 log CFU/g. The final population on pine nuts was 6.59±0.43 log CFU/g, which was significantly higher than any of the other three commodities.

At 45% RH (Fig 1B), the *L*. *monocytogenes* populations decreased significantly after only 1 d of storage on both chickpeas and black pepper (8.21±0.38 and 8.89±0.18 log CFU/g, respectively). The levels of the pathogen on chickpeas and black pepper continued to decrease with populations at 21 d of 6.77±0.41 and 7.28±0.21 log CFU/g, respectively; after 180 d storage, populations decreased to 2.58±0.64 and 1.90±0.37 log CFU/g, respectively. *L*. *monocytogenes* appeared to survive better on both sesame seeds and pine nuts, as was also determined at 25% RH. On sesame seeds, the pathogen significantly decreased after 14 d to 8.63±0.22 log CFU/g and the final population was 4.77±0.12 log CFU/g. Similarly, for pine nuts, *L*. *monocytogenes* significantly decreased after 14 d storage to 8.63±0.22 log CFU/g. The highest final population out of the four commodities was observed after 180 d on pine nuts (6.36±0.19 log CFU/g).

During storage at 75% RH (Fig 1C), the *L*. *monocytogenes* populations decreased significantly after only 1 d of storage on chickpeas, sesame seeds, and black pepper to 8.16±0.44, 8.12±0.32, and 8.47±0.33 log CFU/g, respectively. The final population of the pathogen at 180 d on sesame seeds was 3.41±0.28 log CFU/g. Whereas for chickpeas and black pepper, *L*. *monocytogenes* was below the level of enumeration after 180 d yet was detected in all 1-g samples through enrichment. The populations after 120 d storage on chickpeas and black pepper were 2.68±0.53 and 2.28±0.33 log CFU/g, respectively. On pine nuts, the *L*. *monocytogenes* population remained at 8–9 log CFU/g throughout 60 d storage before significantly decreasing to 7.04±0.27 log CFU/g after 90 d storage. The final level of *L*. *monocytogenes* after 180 d was 3.37±0.43 log CFU/g.

**Fig 2:**
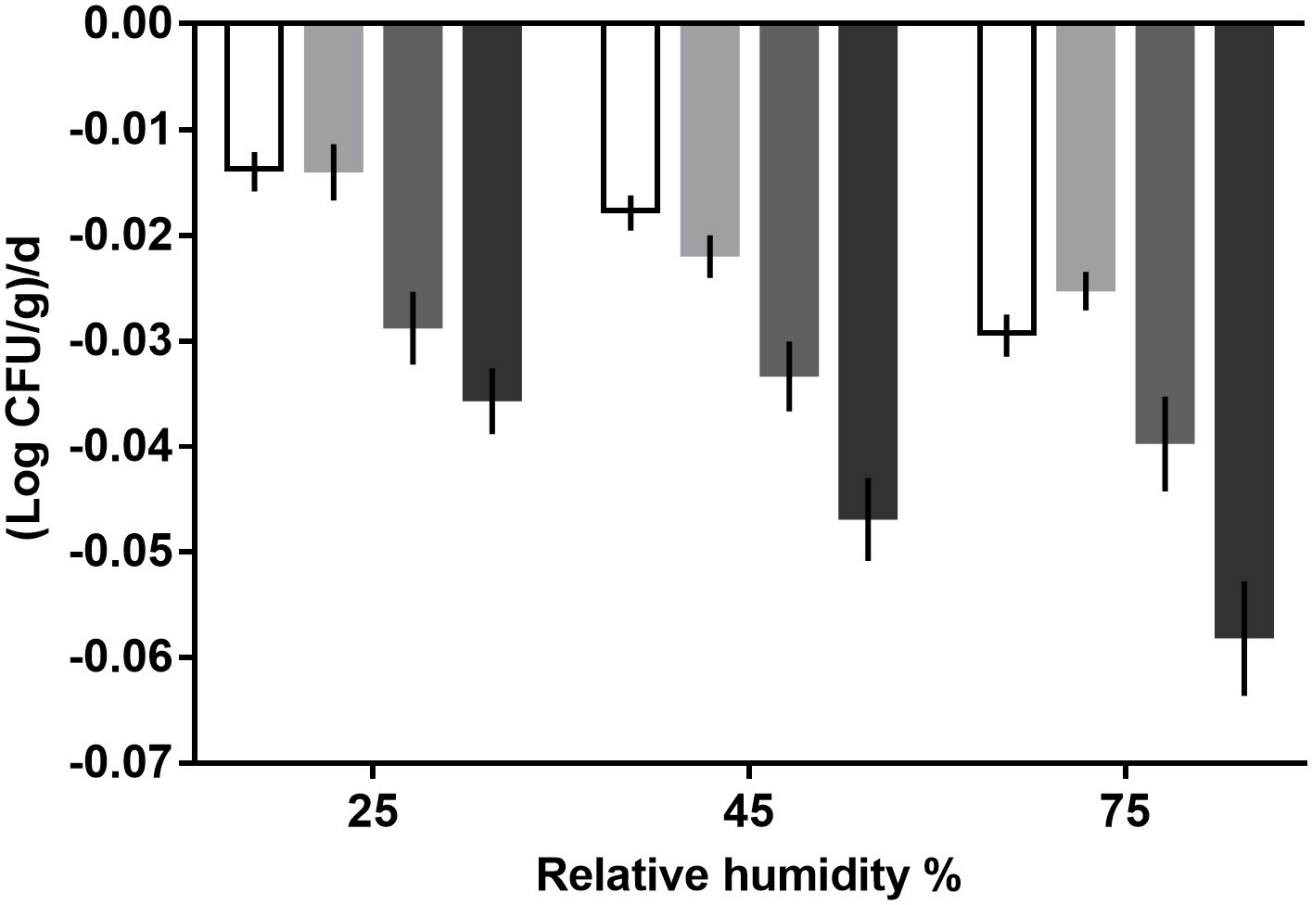
Decay rates of *L*. *monocytogenes* on pine nuts (white bar), sesame seeds (light gray bar), chickpeas (dark gray bar), and black pepper (black bar) during storage at 25°C and 25, 45, or 75% relative humidity. Data points and error bars represent linear regression slope estimates and 95% confidence intervals.

The survival of *L*. *monocytogenes* on the four commodities was highest at 25% RH and lowest at 75% RH (Fig 2). At 25%, the decay rate of *L*. *monocytogenes* was lowest on both pine nuts (-0.014±0.001 log CFU/g per d) and sesame seeds (-0.014± 0.001 log CFU/g per d). The decay rate of the pathogen was the highest on black pepper (-0.036± 0.002 log CFU/g per d). Similar results were observed at both 45 and 75% RH. At 45% RH, the lowest and highest *L*. *monocytogenes* decay rates were observed on pine nuts (-0.018±0.001 log CFU/g per d) and black pepper (-0.047±0.002 log CFU/g per d), respectively. At 75% RH, the lowest and highest decay rates were determined on sesame seeds (-0.025±0.001 log CFU/g per d) and black pepper (-0.058±0.003 log CFU/g per d), respectively. Pairwise comparison of statistical significance for *L*. *monocytogenes* decay rates for all four commodities and three RH levels is presented in S1 Fig. Overall, the survival data presented in this study can be used in risk assessment and in the development of predictive models of *L*. *monocytogenes* survival on these food commodities at various RH levels.

This study determined that *L*. *monocytogenes* survived best on pine nuts and sesame seeds, followed by chickpeas, and finally black pepper. Previous studies have determined similar population dynamics and decay rates for *L*. *monocytogenes* on nuts [22–24]. Brar P.K. et al observed during a 7-month storage on almonds and pistachios at 24°C and 38–39% RH, the decay rates of *L*. *monocytogenes* were -0.024 and -0.029 log CFU/g per d, respectively [22]. On peanuts and pecans stored at 22°C, the decay rates of the pathogen during 1-year storage at 56±8% RH were -0.019 and -0.038 log CFU/g per d [23]. Additionally, on walnuts, the decay rate of *L*. *monocytogenes* during 105 d storage at 23°C and 25–35% RH was -1.1 log CFU/g per month [24]. It is however noted that different *L*. *monocytogenes* strains were used and the experimental conditions were not identical in these published studies. Despite these differences, *L*. *monocytogenes* appears to demonstrate lower decay rates on pine nuts, sesame seeds, and peanuts.

### Survival of *L*. *monocytogenes* as determined by food commodity characteristics

This study determined that *L*. *monocytogenes* survives significantly longer on pine nuts and sesame seeds as compared with chickpeas and black pepper, regardless of RH level (Fig 2 and S1 Fig). The four food commodities selected in this study, in addition to being from different food categories, i.e. legumes, seeds, and spices, were chosen also based on varying physiochemical properties including fat content, general surface topographical characteristics, and antimicrobial constituents. Pine nuts are approximately 50–66% fat (of which oleic and linoleic acids account for more than 85%) and 13–32% protein [26, 33, 34]. Similarly, the fat content of sesame seeds is approximately 55% with a protein content of 20% [35]. Chickpeas and black pepper contain much less fat (3–6% [36] and <1%, respectively). Research suggests that *L*. *monocytogenes* survives longer on food products with higher fat contents [37]; therefore, the higher fat content of the pine nuts and sesame seeds may have aided in the survival of *L*. *monocytogenes*.

## Conclusion

In summary, this study contributes knowledge on the survival of *L*. *monocytogenes* on chickpeas, pine nuts, sesame seeds, and black pepper. This is the first study assessing *L*. *monocytogenes* survival kinetics on a legume, seed, or spice during storage. The results collected herein can aid in understanding how this pathogen survives on these food commodities and the data generated can be used in predictive modeling and risk assessment of *L*. *monocytogenes*.

## Supporting information

S1 Fig Pairwise comparison heatmap of statistical differences for *L*. *monocytogenes* decay rates for pine nuts, sesame seeds, chickpeas, and black pepper at 25, 45, and 75% RH. P-values less than <0.05 are highlighted in grey.
(DOCX)

## References

[1] Harris LJ, Yada S, Beuchat LR. Outbreaks of foodborne illness associated with the consumption of tree nuts, peanuts, and sesame seeds 2018. http://ucfoodsafety.ucdavis.edu/Nuts_and_Nut_Pastes/.

[2] CDC. Multistate outbreak of human Salmonella Enteritidis infections linked to Turkish pine nuts 2011. https://www.cdc.gov/salmonella/2011/pine-nuts-11-17-2011.html.

[3] CDC. Multistate outbreak of Salmonella Montevideo and Salmonella Senftenberg infections linked to Wonderful Pistachios (final update) 2016. https://www.cdc.gov/salmonella/montevideo-03-16/.

[4] CDC. Multistate outbreak of human Salmonella Montevideo infections (final update) 2010. https://www.cdc.gov/salmonella/2010/montevideo-5-4-2010.html.

[5] Zweifel C, Stephan R. Spices and herbs as source of *Salmonella*-related foodborne disease. Food Res Int. 2011;45(2):765–9.

[6] FDA. Gomarco recalls limited number of Macrobars and Thrive bars because of possible health risk 2017. https://www.fda.gov/Safety/Recalls/ucm563265.htm.

[7] FDA. NOW Health Group Inc. expands voluntary recall of Ellyndale Nutty Infusions because of possible health risk 2017. https://www.fda.gov/Safety/Recalls/ucm563399.htm.

[8] FDA. Recall on Fewer Than 225 Ava’s Brand Organic Cashews roasted and salted 8 oz. tubs in NJ, NY, PA, and CT 2017.https://www.fda.gov/Safety/Recalls/ucm560733.htm.

[9] FDA. Kroger recalls Simple Truth dry roasted macadamia nuts because of possible health risk 2017. https://www.fda.gov/Safety/Recalls/ucm560931.htm.

[10] FDA. U.S. House of Thaller recalls selected pine nut hummus products because of possible health risk 2017. https://www.fda.gov/Safety/Recalls/ucm563822.htm.

[11] FDA. Publix recalls cranberry nut and seed mix due to possible health risk 2016. https://www.fda.gov/Safety/Recalls/ucm500558.htm.

[12] FDA. United Natural Trading LLC announces voluntary recall of walnuts 2016. https://www.fda.gov/Safety/Recalls/ucm500345.htm.

[13] News Desk. Illinois firm recalls shelled walnuts for potential Listeria 2014. https://www.foodsafetynews.com/2014/05/illinois-firm-recalls-shelled-walnuts-for-potential-listeria/#.U4TUJC-pVWs.

[14] News Desk. Walnuts recalled due to potential Listeria contamination 2014. https://www.foodsafetynews.com/2014/05/walnuts-recalled-due-to-potential-listeria-contamination/#.U4TUiC-pVWs.

[15] News Desk. Phoenix company announces walnut recall, names California disbributor 2014. https://www.foodsafetynews.com/2014/05/phoenix-firm-announces-walnut-recall-names-california-distributor/.

[16] Beach C. Snacks, nuts, protein bars recalled for Listeria concerns 2016. https://www.foodsafetynews.com/2016/05/snacks-nuts-protein-bars-recalled-for-listeria-concerns/.

[17] Picha D, Pyzner J. Storage hints for pecans. Louisiana State University Agricultural Center. 2013. https://www.lsuagcenter.com/NR/rdonlyres/B4948FCF-C7FA-41F2-9536-8F450B7AF058/20289/STORAGEHINTSFORPECANS.pdf.

[18] Kader AA. Chapter 2: Impact of nut postharvest handling, de-shelling, drying and storage on quality In: Harris LJ, editor. Improving the Safety and Quality of Nuts. Cambridge, UK: Woodhead Publishing Limited; 2013.

[19] Kenney SJ, Beuchat LR. Survival, growth, and thermal resistance of *Listeria monocytogenes* in products containing peanut and chocolate. J Food Prot. 2004;67(10):2205–11. 10.4315/0362-028x-67.10.2205 15508631

[20] Koseki S, Nakamura N, Shiina T. Comparison of desiccation tolerance among *Listeria monocytogenes*, *Escherichia coli* O157:H7, *Salmonella enterica*, and *Cronobacter sakazakii* in powdered infant formula. J Food Prot. 2015;78(1):104–10. 10.4315/0362-028X.JFP-14-249 25581184

[21] Brackett RE, Beuchat LR. Survival of *Listeria monocytogenes* in whole egg and egg yolk powders and in liquid whole egg. Food Microbiol. 1991;8(4):331–7.

[22] Kimber MA, Kaur H, Wang L, Danyluk MD, Harris LJ. Survival of *Salmonella*, *Escherichia coli* O157:H7, and *Listeria monocytogenes* on inoculated almonds and pistachios stored at -19, 4, and 24 degrees C. J Food Prot. 2012;75(8):1394–403. 10.4315/0362-028X.JFP-12-023 22856562

[23] Brar PK, Proano LG, Friedrich LM, Harris LJ, Danyluk MD. Survival of *Salmonella*, *Escherichia coli* O157:H7, and *Listeria monocytogenes* on raw peanut and pecan kernels stored at -24, 4, and 22 degrees C. J Food Prot. 2015;78(2):323–32. 10.4315/0362-028X.JFP-14-327 25710147

[24] Blessington T, Mitcham EJ, Harris LJ. Survival of *Salmonella enterica*, *Escherichia coli* O157:H7, and *Listeria monocytogenes* on inoculated walnut kernels during storage. J Food Prot. 2012;75(2):245–54. 10.4315/0362-028X.JFP-11-278 22289584

[25] Blessington T, Theofel CG, Mitcham EJ, Harris LJ. Survival of foodborne pathogens on inshell walnuts. Int J Food Microbiol. 2013;166(3):341–8. 10.1016/j.ijfoodmicro.2013.07.016 24026009

[26] Farakos SM, Pouillot R, Keller SE. *Salmonella* Survival Kinetics on Pecans, Hazelnuts, and Pine Nuts at Various Water Activities and Temperatures. J Food Prot. 2017;80(5):879–85. 10.4315/0362-028X.JFP-16-392 28414256

[27] Farakos SM, Pouillot R, Anderson N, Johnson R, Son I, Van Doren J. Modeling the survival kinetics of *Salmonella* in tree nuts for use in risk assessment. Int J Food Microbiol. 2016;227:41–50. 10.1016/j.ijfoodmicro.2016.03.014 27062527

[28] Farakos SM, Frank JF, Schaffner DW. Modeling the influence of temperature, water activity and water mobility on the persistence of *Salmonella* in low-moisture foods. Int J Food Microbiol. 2013;166(2):280–93. 10.1016/j.ijfoodmicro.2013.07.007 23973840

[29] Hwang CA, Porto-Fett AC, Juneja VK, Ingham SC, Ingham BH, Luchansky JB. Modeling the survival of *Escherichia coli* O157:H7, *Listeria monocytogenes*, and *Salmonella* Typhimurium during fermentation, drying, and storage of soudjouk-style fermented sausage. Int J Food Microbiol. 2009;129(3):244–52. 10.1016/j.ijfoodmicro.2008.12.003 19157610

[30] Fleming DW, Cochi SL, MacDonald KL, Brondum J, Hayes PS, Plikaytis BD, et al Pasteurized milk as a vehicle of infection in an outbreak of listeriosis. N Engl J Med. 1985;312(7):404–7. 10.1056/NEJM198502143120704 3918263

[31] FDA. Bacteriological Analytical Manual (BAM) Chapter 10: Detection of Listeria monocytogenes in foods and environmental samples, and enumeration of Listeria monocytogenes in foods 2017. https://www.fda.gov/Food/FoodScienceResearch/LaboratoryMethods/ucm071400.htm.

[32] Beuchat LR. Relationship of water activity to moisture content in tree nuts. J Food Sci. 1978;43:754–8.

[33] Venkatachalam M, Sathe SK. Chemical composition of selected edible nut seeds. J Agric Food Chem. 2006;54(13):4705–14. 10.1021/jf0606959 16787018

[34] Nergiz C, Donmez I. Chemical composition and nuritive value of *Pinus pinea* L. seeds. Food Chem. 2004;86:365–8.

[35] Martinchik AN. Nutritional value of sesame seeds. Vopr Pitan. 2011;80(3):41–3. 21842753

[36] Jukanti AK, Gaur PM, Gowda CL, Chibbar RN. Nutritional quality and health benefits of chickpea (Cicer arietinum L.): a review. Br J Nutr. 2012;108 Suppl 1:S11–26. 22916806 10.1017/S0007114512000797

[37] Barmpalia-Davis IM, Geornaras I, Kendall PA, Sofos JN. Effect of fat content on survival of *Listeria monocytogenes* during simulated digestion of inoculated beef frankfurters stored at 7 degrees C. Food Microbiol. 2009;26(5):483–90. 10.1016/j.fm.2009.02.011 19465244

